# Detailed assessment of night shift work aspects and potential mediators of its health effects: the contribution of field studies

**DOI:** 10.3389/fpubh.2025.1578128

**Published:** 2025-05-22

**Authors:** Tara van der Grinten, Daniella van de Langenberg, Linda van Kerkhof, Barbara N. Harding, Anne Helene Garde, Caisa Laurell, Roel Vermeulen, Susan Peters, Jelle Vlaanderen

**Affiliations:** ^1^Institute for Risk Assessment Sciences, Utrecht University, Utrecht, Netherlands; ^2^Centre for Health Protection, National Institute for Public Health and the Environment (RIVM), Bilthoven, Netherlands; ^3^Department of Non-Communicable Diseases and Environment, Barcelona Institute of Global Health (ISGlobal), Barcelona, Spain; ^4^Universitat Pompeu Fabra (UPF), Barcelona, Spain; ^5^CIBER Epidemiología y Salud Pública (CIBERESP), Madrid, Spain; ^6^The National Research Centre for the Working Environment (NFA), Copenhagen, Denmark; ^7^Unit of Occupational Medicine, Institute of Environmental Medicine (IMM), Karolinska Institutet, Stockholm, Sweden

**Keywords:** occupational health, circadian rhythm, risk factors, sleep, light exposure, work schedule tolerance

## Abstract

Night shift work has been associated with adverse health outcomes, but inconsistencies in epidemiological findings reveal gaps in understanding the mechanisms involved. Beyond shift schedules (e.g., duration and intensity) and nighttime light exposure, we propose assessing ten key aspects to enhance understanding of shift work’s nature and health implications. These include: (1) exposure-related factors (“meal timing and composition during the night shift,” “physical activity during the night shift”); (2) potential mediators (“supplements and medication use,” “social disruption,” “sunlight exposure,” “meal timing and dietary patterns outside shifts,” “physical activity,” “sleep quality,” and “substance use”); and (3) effect modifiers (“occupational co-exposures”). Recent advances in technology, such as mobile apps, wearable sensors, and biomarkers, enable real-time, multidimensional assessments of these factors in field studies. Incorporating these tools into high-quality data collection can provide critical insights into the pathways linking night shift work and health. Such approaches will generate new hypotheses and inform the design of next-generation cohort and case–control studies, fostering a deeper understanding of this complex exposure and its health implications.

## Introduction

Exposure to night shift work is associated with an increased risk of chronic diseases, including metabolic syndrome and cancer. However, existing epidemiological studies often yield inconsistent findings regarding these risks and the pathways through which night shift work impacts health.

Stevens et al. attributed the lack of consistency of findings in the epidemiological (cancer) literature to the varying quality of exposure assessment that was used to characterize night shift work in these studies (1). To address this, they proposed a more accurate and comprehensive approach in future studies, which would include assessing factors such as working hours, the duration and intensity of night shifts, rotation types, rest periods, sleep quality, and individual characteristics like chronotype. Additionally, they recommended exploring how these factors might modify the effects of night shift work on health.

In a recent publication, Papantoniou and Hansen emphasized the importance of detailed exposure assessments, noting that case–control studies are better suited than cohort studies for this purpose (2). Some recent cohorts have started incorporating similar detailed exposure assessments (3).

We argue that, in addition to the statements made by Stevens and Papantoniou, assessing additional aspects directly or indirectly related to night shift work could further enhance our understanding of what night shift work is and how it affects day-to-day life. This can lead to better insights into the development of chronic disease. While it is crucial to accurately describe the exposure night shift work itself, the assessment of factors that potentially mediate the effects of night shift work on health, and the assessment of factors for which night shift work is a potential effect modifier is of equal importance.

We argue that, beyond the factors highlighted by Stevens and Papantoniou, further examination of aspects related to night shift work could deepen our understanding of its impact on health. This includes not only accurately describing the exposure itself but also considering mediators and effect modifiers of its health effects.

Accurately assessing these factors requires a suite of exposure assessment tools. While cohort and case–control studies rely heavily on questionnaires, cross-sectional, panel, or short-term longitudinal studies allow for the inclusion of objective measurements. These studies can play a crucial role in identifying potential mediators and informing intervention strategies, which is especially important given the inevitability of night shift work in modern society.

**Figure 1:**
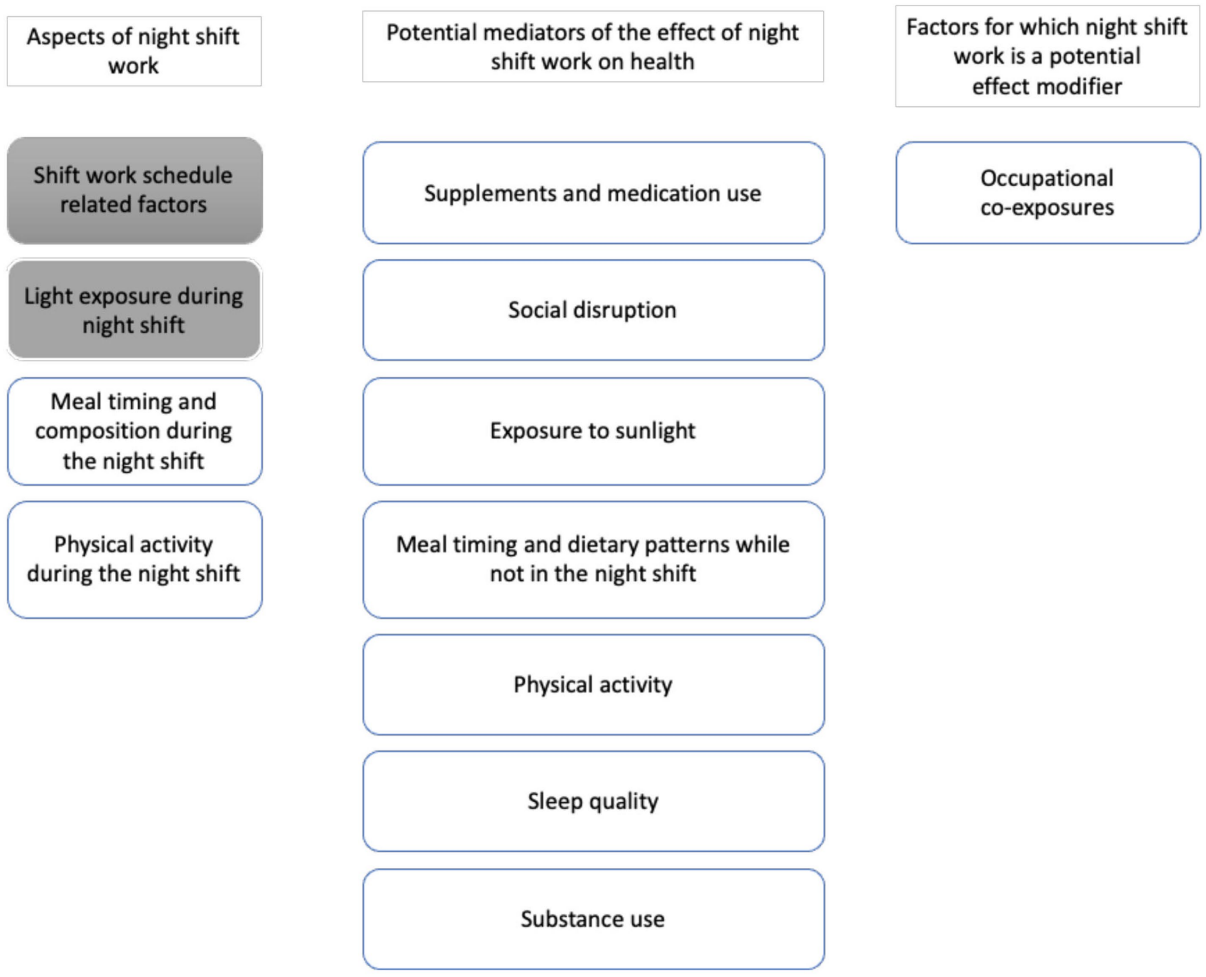
Overview of aspects of night shift work, potential mediators, and factors for which night shift work is a potential effect modifier that can be assessed in a field study. Shaded factors have been described previously in Stevens et al. (1).

We propose a novel framework for understanding the diverse factors involved in night shift work exposure and its health implications (Figure 1). This figure provides a graphical overview of the key aspects that should be considered, including both the core components of shift work and potential effect modifiers, which we believe will help guide future research in this field.

## Aspects that are part of night shift work

Exposure to night shift work is a complex mixture of factors (4). These exposures can disturb the circadian rhythm, which might lead to chronic health effects. We consider four aspects that are part of the shift work exposure mixture. Two shiftwork-related aspects were included in Stevens et al. (1): ‘shift work schedule related factors’ and ‘light exposure during the night shift’. We propose to consider two additional aspects as part of the exposure to night shift work: “Meal timing and composition during the night shift” and “Physical activity during the night shift.”

### Shift work schedule-related factors

Employer registry, shift schedules, and payroll data can be used to assess working schedules in a study population (5). While such data provides objective information, it is limited in the sense that it does not provide information on unpaid working hours, deviations from the schedule, etc. To fill these gaps in exposure assessment, in studies combinations of methods such as questionnaires, interviews, and diary methods can be applied. In addition, day-to-day logging of the work schedule can be applied for a detailed and unbiased assessment of the shift work schedule. To alleviate the considerable efforts that this would require from study participants, smartphone-based applications can improve user-friendliness and accessibility when implemented correctly (6).

### Light exposure during the night shift

In cohort studies, exposure to light can be assessed using a questionnaire. Examples include recording the light source to which one is exposed hourly for seven consecutive days (7) or more specific questions focused on light circumstances at work during the biological night (3). However, different light sources can result in different light (spectral) exposures. To be assess the exposure, lighting conditions can also be verified more precisely using UV-sensitive badges or light sensors, which record light intensity. Ideally, light sensors would be able to differentiate between wavelengths, enabling the assessment of blue light specifically. Blue light is hypothesized to be most important in entraining the circadian pacemaker and can therefore lead to the most circadian disruption (8). The level of exposure to blue light varies between light sources, with artificial light as a major source of it. While sensor data is more accurate, the implementation of sensors is cumbersome for study participants, or not allowed in certain settings for safety reasons, and can be expensive. The use of ambient light sensors on smartphones to assess light exposure is a potential avenue. However, the reliability of such measurements is currently still low (9).

### Meal timing and composition during the night shift

Meal timing and composition are part of the night shift work exposure mixture. For example, one could envisage that a night shift during which healthy food is provided, and workers can consume their food at a time closest to the time they would consume their food when not working in a night shift is less impactful on health than a night shift which is characterized by consuming (unhealthy) food at times completely out of sync with their circadian rhythm. It is therefore important to accurately assess these aspects and incorporate them as one of the dimensions relevant for the exposure assessment of night shift work.

Questionnaires such as the Food-Based Classification of Eating Episodes (FBCE) (10) are commonly used to assess meal timing and composition in cohort studies. In short-term studies, 24-h recall questionnaires that provide more detailed and potentially less-biased exposure assessment can be implemented (11). For a representative assessment, these 24-h recall questionnaires should ideally be repeated multiple days (12). Even more detailed and less biased assessment of food intake is possible through the use of mobile phone applications that allow for the prolonged and real-time collection of dietary information, especially when combined with food photographs taken by study participants to reduce measurement error (13). Furthermore, new developments are focused on combining food photography with artificial intelligence to increase precision and decrease the workload of the analyses (14).

### Physical activity during the night shift

The degree of physical activity during the night shift is part of the night shift work exposure mixture as night shifts characterized by strenuous physical activity can be envisaged to be perturbing the circadian rhythm more than night shifts that are characterized by sedentary activities. It is therefore important to accurately assess this aspect and incorporate it as one of the dimensions relevant for the exposure assessment of night shift work.

For cohort studies, questionnaires such as the International Physical Activity Questionnaire (IPAQ) are available to assess physical activity (15). In short-term studies, data from questionnaires can be supplemented with more objective and precise measurements through the use of actigraphy sensors, especially research-grade sensors (11, 16). The costs of research-grade sensors will have to decrease to make them available for larger studies.

## Potential mediators of the effect of night shift work on health

As the precise mechanisms through which night shift work impacts health are not yet fully understood, it is crucial to better understand the factors that may mediate the health effects of night shift work. High-quality assessment of these potential mediators will help elucidate their role in the pathway linking night shift work exposure to health outcomes and provide potential targets for intervention. We propose to consider seven potential mediators of the effect of night shift work on health.

### Supplements and medication use

Due to light exposure and the decrease in sleep quality, night shift workers might choose to use over-the-counter supplements and prescription medicine such as melatonin, Vitamin D, or sleep medication (17, 18). Questionnaires and registry data have been used in cohort studies to assess supplement and medication use (19, 20). In short-term studies, the collection of biospecimens and the use of biomarkers would allow for objective assessment of supplement and medication use, especially when also incorporating issues such as adherence to prescription, absorption, distribution, metabolism, and excretion. The drug metabolites could be identified through methods like high-resolution mass-spectometry (HRMS) (21).

### Social disruption

Night shift work has been reported to have an impact on social disruption (22). In cohort studies disturbed social patterns can be assessed using questionnaires such as the Standard Shift Work Index (SSI) including the Social and Domestic Disruption Scale.

In short-term studies, it is also possible to interview participants, which is more time-consuming yet might provide more detailed information. This qualitative approach can provide a more nuanced understanding of the social dynamics, coping strategies, and impact of night shift work on social behavior. Another avenue is the use of smartphone applications to collect data on social behavior by measuring social aspects such as phone calls, interactions (through Bluetooth), and movement patterns. Apps using Bluetooth to study interactions have been used during the covid pandemic for contact tracing (23). These applications require further development before they can generate reliable insights (24).

### Exposure to sunlight

In addition to an increased exposure to light at night, being active in night shift work can also have an impact on exposure to sunlight during the day (25). Compared to day workers, night shift workers can spend more time in natural daylight during their time off, but also receive more artificial light exposure during their night shifts (11).

Methods to assess exposure to sunlight are the same as those mentioned to assess light exposure during the night shift. In addition, questionnaires can assess the time spend outdoors during the day (11). Sensors can also be used for 24 h logging. However, requiring a study participant to wear a sensor for 24 h a day can be quite cumbersome. A wrist-worn sensor might therefore be more user-friendly to the participant but comes with the risk of less reliable results than sensors worn close to the eyes.

### Meal timing and dietary patterns while not on the night shift

Diet is influenced by both the work schedule as well as social obligations, which can lead to sub-optimal meal timing (11, 26), energy intake, and diet composition (27).

While the methods to assess meal timing and dietary are the same as those mentioned during the night shift, it may pose a larger burden on participants to continue this during the day. For accurate assessment of long-term food intake patterns, 24-h recall questionnaires should be repeated on multiple days and therefore require considerable effort from study participants (12).

### Physical activity

There is conflicting evidence on the impact of night shift work on physical activity outside working hours. Some studies report an increase while others report a decrease in physical activity (11, 28, 29). Therefore, more personalized measurements are needed. Not only to assess the exposure but also to explore potential determinants that may explain the diverse observation on PA and shift work to date.

The same methods to assess physical activity during the night shift can be used during the day. Manually logging activity can ask more time than during the night shift, due to a higher variety of sports and other activities.

### Sleep quality

Working shifts has an impact on both sleep duration and sleep quality-related downstream effects on the circadian rhythm through social jetlag (30, 31). Commonly used methods to assess sleep in large-scale population studies include subjective measures (questionnaires and diaries) such as the Medical Outcomes Study sleep scale (32), the Karolinska Sleep Questionnaire (33), and the Pittsburgh Sleep Quality Index (34).

In short-term studies, actigraphy sensors can be used to measure sleep duration and quality more objectively. Limitations of actigraphy include the inability to distinguish sleep from inactivity and a lack of insight into sleep architecture (sleep stages, latencies, and bio-physiological changes within sleep) (35). Such insights could be acquired through the use of electroencephalography-based methods (36). Interesting novel approaches to assess sleep quality could include the use of (commercial) sleep-tracking apps. However, these need to be further developed as currently available applications typically underestimate sleep disruption and overestimate sleep duration and efficiency (37, 38).

### Substance use

Working in night shifts has been reported to affect substance use such as illicit drugs (39), alcohol consumption (40), smoking (41, 42), and consumption of caffeine (43).

In cohort studies, substance use can be assessed using questionnaires (44). In short-term studies, more detailed information can be collected by asking study participants to keep a log during the observational period collecting information on substance use. Smartphone applications can also be used to monitor aspects related to everyday behavior (like alcohol usage and smoking) in a cost-effective way (45). Similar to the assessment of supplements and prescription drugs, the collection of biospecimens and the use of biomarkers will also allow for objective assessment of substance use.

## Factors for which night shift work is a potential effect modifier

### Occupational co-exposures

Not only does night shift work include multiple occupations that bring a higher risk of hazardous co-exposures like carcinogens, but studies have also shown that night shift workers have higher levels of exposure to hazards like dust and solvents than their day-working colleagues (46, 47). Night shift work may act as an effect modifier of the potential health effects of these occupational exposures. For example, due to the changes in metabolism caused by circadian disruption, shift workers may be particularly vulnerable to hazardous exposures (48).

In addition, psychosocial and ergonomic work environment has been shown to differ at different times of the day, with increased risk of injury and psychosocial stressor for shift workers (49–51).

Assessment of co-exposures can be difficult, as the type of co-exposure can vary widely between occupations (4). Most cohort studies use some type of questionnaire or survey to assess co-exposure (46). Job exposure matrices (JEMs) are also often used for occupational co-exposures (52). In field studies, more detailed assessments such as personal sampling of chemical exposures using sensors, wristbands, or biospecimens can be used for objective assessment of chemical co-exposures. In addition, psychosocial work environment such as quantitative and emotional demands can be assessed using smart phone applications and ergonomic work environment such as sitting, standing and walking can be assessed by use of actigraphy.

## Discussion

We argue that field studies can enhance the understanding of night shift work’s impact on health by providing detailed assessments of related aspects. These studies can either directly link exposure to health behaviors, biological changes, or health outcomes, or indirectly calibrate and impute exposures in larger case–control or cohort studies. We identify three key contributions of short-term studies with high-quality exposure assessments:

### Hypothesis generation

Short-term studies cannot establish associations between night shift work-related aspects and disease but can generate hypotheses about which aspects may be most disrupted by night shift work and warrant further research. For example, the Klokwerk study observed differences in diet, physical activity, and daylight exposure between night-shift and day workers, emphasizing the importance of time-resolved measurements in night shift work research (11). The results highlighted the importance of time-resolved measurements in night shift work research.

### Validation and calibration of exposure assessment

High-quality exposure data from short-term studies can serve as external validation to improve exposure assessments in larger epidemiological studies with lower quality data. While this approach has not been widely applied in night shift work research, its potential is highlighted by studies in other areas. For example, Hall et al. (53) demonstrated how the precision of exposure assessment influenced the observed relationship between night shift work and depression. Similarly, Reedijk et al. (54) successfully applied regression calibration for exposure assessment, combining operator-recorded data with self-reported mobile phone use data.

#### Contribution to the exploration of causal pathways

Short-term studies are limited in their ability to establish causal inference, but they can still provide valuable insights into potential causal pathways. By examining associations between night shift work exposure factors and intermediates on the pathway to health outcomes, these studies can help develop hypotheses about the mechanisms through which night shift work may impact health. Although causal conclusions cannot be drawn, short-term studies can guide future research by identifying key factors for more in-depth exploration. Most existing studies on night shift work have focused on a narrow set of exposure factors, so comprehensive screening studies—potentially including qualitative research—are needed to assess all relevant aspects of exposure. Based on the findings from these studies, hypotheses can be developed and tested in follow-up studies. Existing frameworks that propose potential mechanisms through which night shift work could affect health (55) should be expanded to include the full range of factors discussed here.

## Practical considerations

Night shift work already places a heavy burden on workers, and assessing all the exposure factors described here would require complex study protocols. We advocate for minimally invasive and self-collection methods, which have proven effective in several studies, particularly during the COVID-19 pandemic (56, 57). These methods allow easier integration of participation into workers’ daily routines. While quality is key, lower-quality assessments can be compensated for by larger sample sizes.

Simultaneously incorporating multiple night shift work-related factors in a single study presents statistical challenges. Approaches such as variable selection, dimension reduction, and network modeling can improve inference in epidemiological studies and are applicable to night shift work research (58).

To optimally assess these factors, a balance should be struck between minimizing bias and avoiding overly complex or costly methods. Combining questionnaires with objective measurements via sensors can help reduce bias while keeping participant burden low. Practical constraints such as equipment availability, data integration, and institutional requirements should be considered carefully in the study design. For example, in some settings wearable sensors may not be feasible due to cost or safety policies. Engagement with occupational health services or worker organizations may improve study participation and retention.

## Conclusion

We have highlighted key aspects of night shift work and emphasized the importance of high-quality exposure assessment in field studies. Incorporating advanced exposure tools, such as mobile apps, sensors, and biomarkers, can enhance our understanding and generate hypotheses for future cohort and case–control studies. Beyond research applications, the proposed framework may also inform workplace policies, guide the identification of potential intervention targets to reduce health risks among night shift workers. Although developed with night shift work in mind, many of its components are relevant to other forms of irregular or precarious employment. This broadens the framework’s utility for improving worker health across diverse occupational settings.

## Data Availability

The original contributions presented in the study are included in the article/supplementary material, further inquiries can be directed to the corresponding author/s.
